# Rapid typing of foot-and-mouth disease serotype Asia 1 by reverse transcription loop-mediated isothermal amplification

**DOI:** 10.1186/1743-422X-8-489

**Published:** 2011-10-31

**Authors:** Hao-tai Chen, Jie Zhang, Yong-sheng Liu, Xiang-tao Liu

**Affiliations:** 1 State Key Laboratory of Veterinary Etiologic Biology, National Foot-and-Mouth Disease Reference Laboratory of China, Key laboratory of Animal Virology of Ministry of Agriculture, Lanzhou Veterinary Research Institute, Chinese Academy of Agricultural Sciences, Lanzhou, 730046, Gansu, P.R. China

**Keywords:** Foot-and-mouth disease, Serotype Asia 1, Detection, Reverse transcription loop-mediated isothermal amplification

## Abstract

A reverse transcriptase loop-mediated isothermal amplification (RT-LAMP) assay was rapidly used to detect serotype Asia 1 of foot-and-mouth disease virus (FMDV) within 45 min at 61°C. All FMDV serotype Asia 1 reference strains were positive by RT-LAMP, while other viruses such as FMDV serotypes O, C, A and classical swine fever virus, swine vesicular disease virus, porcine reproductive and respiratory syndrome virus and Japanese encephalitis virus remained negative. Furthermore, FMDV sreotype Asia 1 positive samples were able to detect by RT-LAMP assay. This RT-LAMP assay may be suitable particularly for diagnosis of FMDV serotype Asia 1 infection in field stations.

## Article Outline

Foot-and-mouth disease virus (FMDV) is a member of the genus *Aphthovirus *of the family *Picornaviridae*, which is divided into seven serotypes with no cross-protection conferred among the serotypes [1]. FMDV serotypes O, A, C are widely distributed worldwide, whereas FMDV serotypes SAT 1, SAT 2, SAT 3 are normally restricted to Africa and FMDV serotype Asia 1 to Asia [2,3]. Due to the aggressive nature of foot-and-mouth disease (FMD), outbreaks usually result in severe economic losses and impact on both national and international trade within the livestock and animal products [4-6]. Rapid and accurate diagnosis of any suspected FMD cases is of utmost urgency to control this veterinary infection given the extreme contagiousness of the causative virus.

Conventional laboratory diagnosis of FMD was made by enzyme-linked immunosorbent assay (ELISA) detection of specific viral antigens and by observation of cytopathogenic effects in cell culture [4,7-9]. Alternatively, conventional reverse transcriptase polymerase chain reaction (RT-PCR) [5,10-14] and real-time RT-PCR [6,15-18] were developed to complement primary diagnostic techniques for the FMDV infection. These assays were time-consuming and laborious, which required centralized laboratory facilities and clinical specimen submissions, resulted in the delay of FMDV diagnosis. Given these problems, a rapid, simple, and practical assay to detect FMDV in animal and its products was therefore required in clinical practice.

A novel nucleic acid amplification method, termed reverse transcription loop-mediated isothermal amplification (RT-LAMP), which was applied successfully to the detection of influenza A virus, Newcastle disease virus and classical swine fever virus, porcine reproductive and respiratory syndrome virus [19-22]. In this study, RT-LAMP assay was developed a diagnostic method for the typing of FMDV serotype Asia 1, and then the sensitivity and specificity of the RT-LAMP assay were evaluated using the clincal samples.

Four primers including FIP, BIP, F, and B for RT-LAMP were designed by targeting the conserved regions of VP1 sequence from FMDV serotype Asia 1 (Table 1). RT-LAMP was performed in 25 μl of a mixture containing 1 μl of RNA, 25 pmol (each) of primers FIP and BIP, 10 pmol (each) of primers F and B, 1 U of the THERMO-X reverse transcriptase (Invitrogen) and 6 U of Bst DNA polymerase (New England Biolabs) with the corresponding buffer, respectively. Amplification was carried out at the different temperatures. The reaction was then terminated by incubation at 80°C for 2 min.

**Table 1:** Detection results of the RT-LAMP assay using 125 clinical samples.

Pathogen	Strain (specimen number)	Results (positive number/specimen number tested)	
		
		RT-LAMP	RT-PCR
FMDV	O/CHA/1999 (N = 32)	-- (32/32)	+ (32/32)
	A/CHA/2009 (N = 22)	--(22/22)	+ (22/22)
	Asia 1/JS/2005 (N = 20)	+ (20/20)	+ (20/20)
	C/UN/1958 (N = 5)	-- (5/5)	+ (5/5)
CSFV	C2008 (N = 17)	-- (17/17)	-- (17/17)
SVDV	SVDV01 (N = 10)	-- (10/10)	-- (10/10)
PRRSV	HPBEDV (N = 10)	-- (10/10)	-- (10/10)
JEV	JEV2009 (N = 9)	-- (9/9)	-- (9/9)

+, positive reaction; --, negative reaction

To optimise the RT-LAMP assay, the different temperatures 60, 61, 62, 63°C at 25, 35, 45, 55 min were evaluated. Already after 45 min at 61°C a obvious product could be visualised by gel-electrophoresis in the study. Another useful feature of RT-LAMP was that its products can be observed directly by naked eye, because a white precipitate of magnesium pyrophosphate forms in the reaction tube [23]. Adding the chemical dyestuff to LAMP reactions also was able to increase the ease detection by the naked eye [24].

**Figure 1:**
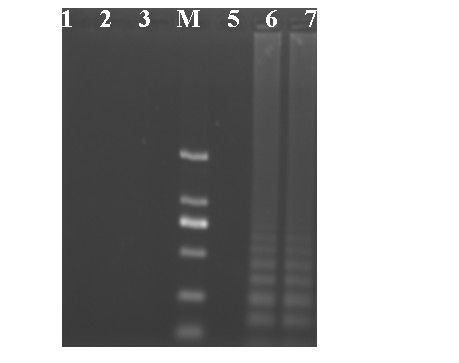
**Agarose gel electrophoresis analysis of the RT-LAMP products using the FMDV reference strains**. Lane 1, O/CHA/2009; Lane 2, A/CHA/2009; Lane 3, C/SU/1958; Lane M, DNA Marker DL2000 (2000, 1000, 750, 500, 250, 100 bp); Lane 5, O/CHA/1999; Lane 6, Asia 1/XJ/2003; Lane 7, Asia 1/JS/2005.

To check that the RT-LAMP reaction was specific for FMDV serotype Asia 1, RNA from reference strains including FMDV serotypes O (O/CHA/1999, O/CHA/2009), A (A/CHA/1972, A/CHA/2009), C (C/SU/1958), Asia 1 (Asia 1/JS/2005) and classical swine fever virus (CSFV), swine vesicular disease virus (SVDV), porcine reproductive and respiratory syndrome virus (PRRSV) and Japanese encephalitis virus (JEV) were tested. The results indicated that the RT-LAMP assay was able to type the FMDV serotype Asia 1 strain used in this experiment as each showed the characteristic ladder-like pattern in the gel (Figure 1). As expected, the other viruses containing FMDV serotypes O, C, A and CSFV, SVDV, PRRSV or JEV gave a negative result by the RT-LAMP assay.

To assess the applicability of this method, 125 clinical samples were used in the study (Table 2). All samples were also identified by RT-PCR, respectively. The details of primers and condition for the RT-PCR assay for the detection of FMDV have been previously described [3]. The result indicated that 20 positive samples of FMDV serotype Asia 1 were typed by RT-LAMP, but 32 FMDV serotype O, 5 FMDV serotype C, 22 FMDV serotype A, 17 CSFV, 10 SVDV, 10 PRRSV, 9 JEV samples gave the negtive in the study.

**Table 2:** Details of RT-LAMP primers designed for detection of the VP1 sequence of FMDV serotype Asia 1.

Primer name	Sequence
F	5'-ACCACAACCACAGGCGAGTC-3'
B	5'-AGCGCAATCTCCAGGTCTGA-3'
FIP	5'-TTCACAAACCTGTCGAGAAC +TTTT+ CCACTACGGTGGAGAACTAC-3'
BIP	5'-ATGCAGATACCCTCACACAC +TTTT+ AAGTAGTACGTCGCAGACCG-3'

Taken together, the RT-LAMP assay was rapid, specific, and sensitive for typing FMDV serotype Asia 1 in clinical samples from the infected pigs. This method not only reduced the diagnosis time significantly but also may be a potential for wider use in field practice.

## Competing interests

The authors declare that they have no competing interests.

## Authors' contributions

HT C and J Z designed the research, then carried out most of the experiments. XT L supported experimets. HT C and YS L wrote and revised the manuscript. All of the authors approved the final version of the manuscript.

## References

[B1] Carrillo C Tulman ER Delhon G Lu Z Carreno A Vagnozzi A Kutish GF Rock DL Comparative genomics of foot-and-mouth disease virus J Virol 2005 79 10 6487 504 10.1128/JVI.79.10.6487-6504.2005 15858032 PMC1091679

[B2] Grubman MJ Baxt B Foot-and-mouth disease Clin Microbiol Rev 2004 17 465 493 10.1128/CMR.17.2.465-493.2004 15084510 PMC387408

[B3] Fernandez J Aguero M Romero L Sanchez C Belak S Arias M Sanchez-Vizcaino JM Rapid and differential diagnosis of foot-and-mouth disease, swine vesicular disease, and vesicular stomatitis by a new multiplex RT-PCR assay J Virol Methods 2008 147 301 311 10.1016/j.jviromet.2007.09.010 17964668

[B4] Ferris NP Dawson M Routine application of enzyme-linked immunosorbent assay in comparison with complement fixation for the diagnosis of foot-and-mouth and swine vesicular diseases Vet Microbiol 1988 16 201 209 10.1016/0378-1135(88)90024-7 3376418

[B5] Reid SM Ferris NP Hutchings GH Samuel AR Knowles NJ Primary diagnosis of foot-and-mouth disease by reverse transcription polymerase chain reaction J Virol Methods 2000 89 167 176 10.1016/S0166-0934(00)00213-5 10996650

[B6] King DP Ferris NP Shaw AE Reid SM Hutchings GH Giuffre AC Robida JM Callahan JD Nelson WM Beckham TR Detection of foot-and-mouth disease virus: comparative diagnostic sensitivity of two independent real-time reverse transcription-polymerase chain reaction assays J Vet Diagn Invest 2006 18 93 97 10.1177/104063870601800114 16566264

[B7] Hamblin C Armstrong RM Hedger RS A rapid enzyme-linked immunosorbent assay for the detection of foot-and-mouth disease virus in epithelial tissues Vet Microbiol 1984 9 435 443 10.1016/0378-1135(84)90064-6 6093338

[B8] Roeder PL Le Blanc Smith PM Detection and typing of foot-and-mouth disease virus by enzyme-linked immunosorbent assay: a sensitive, rapid and reliable technique for primary diagnosis Res Vet Sci 1987 43 225 232 2825310

[B9] Zhang Z Donaldson AI Garland AJ The pathogenesis and diagnosis of foot-and-mouth disease J Comp Pathol 2003 129 1 36 10.1016/S0021-9975(03)00041-0 12859905

[B10] Bao HF Li D Guo JH Lu ZJ Chen YL Liu ZX Liu XT Xie QG A highly sensitive and specific multiplex RT-PCR to detect foot-and-mouth disease virus in tissue and food samples Arch Virol 2008 153 205 209 10.1007/s00705-007-1082-y 17987350

[B11] Amaral-Doel CM Owen NE Ferris NP Kitching RP Doel TR Detection of foot-and-mouth disease viral sequences in clinical specimens and ethyleneimine-inactivated preparations by the polymerase chain reaction Vaccine 1993 11 415 421 10.1016/0264-410X(93)90281-2 8385843

[B12] Callens M De Clercq K Differentiation of the seven serotypes of foot-and-mouth disease virus by reverse transcriptase polymerase chain reaction J Virol Methods 1997 67 35 44 10.1016/S0166-0934(97)00074-8 9274816

[B13] Reid SM Hutchings GH Ferris NP De Clercq K Diagnosis of foot-and-mouth disease by RT-PCR: evaluation of primers for serotypic characterisation of viral RNA in clinical samples J Virol Methods 1999 83 113 123 10.1016/S0166-0934(99)00113-5 10598089

[B14] Vangrysperre W De Clercq K Rapid and sensitive polymerase chain reaction based detection and typing of foot-and-mouth disease virus in clinical samples and cell culture isolates, combined with a simultaneous differentiation with other genomically and/or symptomatically related viruses Arch Virol 1996 141 331 344 10.1007/BF01718403 8634024

[B15] Callahan JD Brown F Osorio FA Sur JH Kramer E Long GW Lubroth J Ellis SJ Shoulars KS Gaffney KL Rock DL Nelson WM Use of a portable real-time reverse transcriptase-polymerase chain reaction assay for rapid detection of foot-and-mouth disease virus, J Am Vet Med Assoc 2002 220 1636 1642 10.2460/javma.2002.220.1636 12051502

[B16] Moniwa M Clavijo A Li M Collignon B Kitching PR Performance of a foot-and-mouth disease virus reverse transcription-polymerase chain reaction with amplification controls between three real-time instruments J Vet Diagn Invest 2007 19 9 20 10.1177/104063870701900103 17459827

[B17] Rasmussen TB Uttenthal A de Stricker K Belak S Storgaard T Development of a novel quantitative real-time RT-PCR assay for the simultaneous detection of all serotypes of foot-and-mouth disease virus, Arch Virol 2003 148 2005 2021 10.1007/s00705-003-0145-2 14551821

[B18] Reid SM Ferris NP Hutchings GH Zhang Z Belsham GJ Alexandersen S Detection of all seven serotypes of foot-and-mouth disease virus by real-time, fluorogenic reverse transcription polymerase chain reaction assay J Virol Methods 2002 105 67 80 10.1016/S0166-0934(02)00081-2 12176143

[B19] Pham HM Nakajima C Ohashi K Onuma M Loop-mediated isothermal amplification for rapid detection of Newcastle disease virus J Clin Microbiol 2005 43 1646 1650 10.1128/JCM.43.4.1646-1650.2005 15814979 PMC1081312

[B20] Poon LLM Leung CSW Chan KH Lee JHC Yuen KY Guan Y Peiris JSM Detection of Human Influenza A Viruses by Loop-Mediated Isothermal Amplification J Clin Microbiol 2005 43 427 430 10.1128/JCM.43.1.427-430.2005 15635005 PMC540134

[B21] Chen HT Zhang J Sun DH Ma LN Liu XT Quan K Liu YS Reverse transcription loop-mediated isothermal amplification for the detection of highly pathogenic porcine reproductive and respiratory syndrome virus J Virol Methods 2008 153 2 266 268 10.1016/j.jviromet.2008.07.006 18706931 PMC7112790

[B22] Chen HT Zhang J Ma LN Ma YP Ding YZ Liu XT Chen L Ma LQ Zhang YG Liu YS Rapid pre-clinical detection of classical swine fever by reverse transcription loop-mediated isothermal amplification Mol Cell Probes 2009 23 2 71 74 10.1016/j.mcp.2008.12.001 19103283 PMC7126361

[B23] Mori Y Nagamine K Tomita N Notomi T Detection of loop-mediated isothermal amplification reaction by turbidity derived from magnesium pyrophosphate formation Biochem Biophys Res Commun 2001 289 150 154 10.1006/bbrc.2001.5921 11708792

[B24] Iwamoto T Sonobe T Hayashi K Loop-Mediated Isothermal Amplification for Direct Detection of Mycobacterium tuberculosis Complex, M. avium, and M. intracellulare in Sputum Samples J Clin Microbiol 2003 41 2616 2622 10.1128/JCM.41.6.2616-2622.2003 12791888 PMC156570

